# Lipid and Non-lipid Factors Affecting Macrophage Dysfunction and Inflammation in Atherosclerosis

**DOI:** 10.3389/fphys.2018.00654

**Published:** 2018-06-26

**Authors:** Mark S. Gibson, Neuza Domingues, Otilia V. Vieira

**Affiliations:** Lysosomes in Chronic Human Pathologies and Infection, Faculdade de Ciências Médicas, Centro de Estudos de Doenças Crónicas, NOVA Medical School, Universidade NOVA de Lisboa, Lisbon, Portugal

**Keywords:** chronic inflammation, atherosclerosis, oxidized lipids, macrophage heterogeneity, lysosome dysfunction

## Abstract

Atherosclerosis is a chronic inflammatory disease and a leading cause of human mortality. The lesional microenvironment contains a complex accumulation of variably oxidized lipids and cytokines. Infiltrating monocytes become polarized in response to these stimuli, resulting in a broad spectrum of macrophage phenotypes. The extent of lipid loading in macrophages influences their phenotype and consequently their inflammatory status. In response to excess atherogenic ligands, many normal cell processes become aberrant following a loss of homeostasis. This can have a direct impact upon the inflammatory response, and conversely inflammation can lead to cell dysfunction. Clear evidence for this exists in the lysosomes, endoplasmic reticulum and mitochondria of atherosclerotic macrophages, the principal lesional cell type. Furthermore, several intrinsic cell processes become dysregulated under lipidotic conditions. Therapeutic strategies aimed at restoring cell function under disease conditions are an ongoing coveted aim. Macrophages play a central role in promoting lesional inflammation, with plaque progression and stability being directly proportional to macrophage abundance. Understanding how mixtures or individual lipid species regulate macrophage biology is therefore a major area of atherosclerosis research. In this review, we will discuss how the myriad of lipid and lipoprotein classes and products used to model atherogenic, proinflammatory immune responses has facilitated a greater understanding of some of the intricacies of chronic inflammation and cell function. Despite this, lipid oxidation produces a complex mixture of products and with no single or standard method of derivatization, there exists some variation in the reported effects of certain oxidized lipids. Likewise, differences in the methods used to generate macrophages *in vitro* may also lead to variable responses when apparently identical lipid ligands are used. Consequently, the complexity of reported macrophage phenotypes has implications for our understanding of the metabolic pathways, processes and shifts underpinning their activation and inflammatory status. Using oxidized low density lipoproteins and its oxidized cholesteryl esters and phospholipid constituents to stimulate macrophage has been hugely valuable, however there is now an argument that only working with low complexity lipid species can deliver the most useful information to guide therapies aimed at controlling atherosclerosis and cardiovascular complications.

## Atherosclerosis involves multiple examples of dysregulated cell function

### Excess lipid loading induces lysosome dysfunction

**Figure 1:**
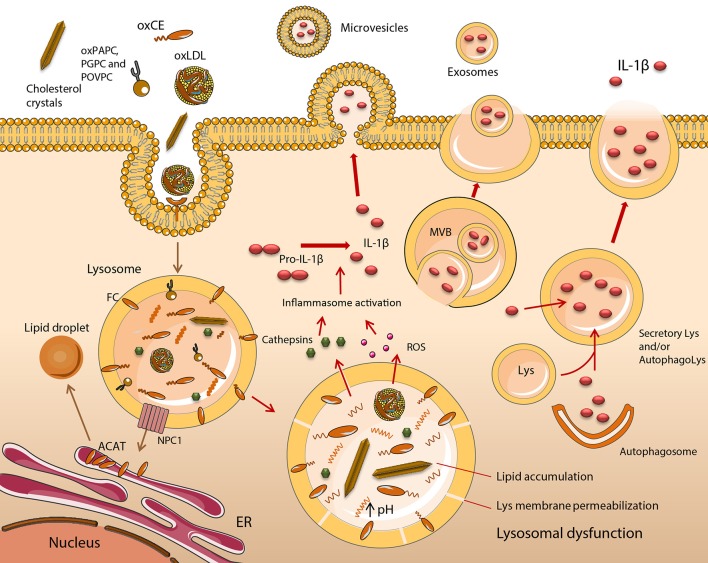
Functional impairment of lipid-engorged lysosomes stimulates inflammasome assembly and IL-1β release. The uptake of lipid and lipoprotein molecules occurs by a variety of scavenger receptor (SR), and Toll-like receptor (TLR)-mediated mechanisms such as phagocytosis or macropinocytosis. In early atherogenesis, monocyte (Mo)-derived macrophages (MOs) retain lipids and lipoproteins, which become degraded in lysosomes, with excess free cholesterol (FC) trafficked to the endoplasmic reticulum (ER). Cholesteryl esters (CE) are hydrolyzed in lysosomes by lysosomal acid lipase (LAL) to FC and fatty acids. FC is trafficked to the ER via Niemann Pick C1 (NPC1), where it becomes esterified by acyl CoA:cholesterol acyltransferase (ACAT), forming CE, which are packaged into cytoplasmic lipid droplets. This is a distinctive feature of foam cell formation. It is induced upon MO cholesterol loading, with lysosomal hydrolysis vital for the mobilization of lipid droplet-associated cholesterol for reverse cholesterol transport. This mechanism of sterol clearance is initially effective, however, when fatty streak lesions degenerate into unstable plaques, a substantial accumulation of CEs, their oxidized derivatives (oxCEs), and FC occurs in lysosomes due to inadequate hydrolysis and clearance. Lysosome dysfunction in excess lipid-loaded MOs is irreversible and is characterized by an inhibition of LAL and cathepsin activity, due to permanent alterations in lysosome function, resulting in the accumulation of cargo. Dysfunctional lysosomes become ruptured, releasing cathepsins, reactive oxygen species (ROS) and other molecules into the cytoplasm. These activate the NLRP3 inflammasome, leading to the maturation and release of IL-1β. Five potential mechanisms of IL-1β release have been described including the exocytosis of secretory lysosomes, exosomes and microvesicle shedding. Lys, lysosome; MVB, multivesicular body.

Atherogenesis occurs when pro-inflammatory monocytes (Mo) are recruited to the intimal layer of the medium and large size arteries. Here, they differentiate into macrophages (MO) and encounter a plethora of modified lipid species such as oxidized low-density lipoprotein (oxLDL). MOs employ mainly scavenger receptors to facilitate the uptake of these modified lipoproteins, leading to the formation of foam cells (Libby et al., 2011). These lipid-rich cells secrete multiple pro-inflammatory mediators that propagate the development of the necrotic core of an atherosclerotic plaque, increasing its vulnerability. This stage also involves significant contributions from the T-cell populations residing in the lesion, however, their involvement in the inflammatory process is beyond the scope of this review and will not be discussed further. Long before this stage is reached, dysregulated lipid metabolism serves to establish the disease. Early atherogenesis involves the development of fatty streaks whereby Mo-derived MOs internalize retained ApoB-containing lipoproteins, which become degraded in lysosomes, with excess free cholesterol (FC) trafficked to the endoplasmic reticulum (ER) (Figure 1). LDL particles ingested via the LDL receptor are degraded and cholesteryl esters (CE) are hydrolyzed in lysosomes to FC and fatty acids. FC is trafficked to peripheral cellular sites by a mechanism involving the proteins Niemann Pick C 1 and 2 (NPC1 & NPC2) (Ouimet et al., 2011) (Figure 1). Delivery of FC to the ER leads to downregulated LDL receptor expression and endogenous cholesterol synthesis through suppression of the sterol-regulatory element-binding protein (SREBP) pathway (Brown and Goldstein, 1997). In the ER, FC is esterified by acyl CoA:cholesterol acyltransferase (ACAT), and the resulting CE is packaged into cytoplasmic lipid droplets, which are a hallmark of foam cells (Brown et al., 1980). Two major pathways facilitate cytoplasmic CE clearance. The first involves the hydrolysis of cytoplasmic CE by cholesterol ester hydrolase (NCEH) and the resulting free cholesterol is mobilized away from the ACAT pool (Brown et al., 1980) and made available for efflux via ATP-binding cassette transporter A1 (ABCA1), scavenger receptor class B type I (SR-BI), and aqueous diffusion. Alternatively, cytoplasmic CEs in lipid droplets are packaged into autophagosomes, which are trafficked to lysosomes, where the CE is hydrolyzed by lysosomal acid lipase (LAL), generating FC for ABCA1-dependent efflux (Ouimet et al., 2011). This process is induced upon MO cholesterol loading, with lysosomal hydrolysis crucial for the mobilization of lipid droplet-associated cholesterol for reverse cholesterol transport (Ouimet and Marcel, 2012). At this stage, lysosomal hydrolysis and sterol clearance is effective. However, the progression of fatty streak lesions to unstable plaques is characterized by the substantial accumulation of CEs and FC in lysosomes (Miller and Kothari, 1969; Fowler et al., 1980; Jerome and Lewis, 1990), indicating a failure to adequately hydrolyse and clear them. This confirms lysosome dysfunction is a key event in late-stage atherosclerotic disease. This phenomenon has been observed *in vivo* and replicated *in vitro*. In pigeons, cholesterol trapped in the lysosomes of lesional foam cells remained trapped, even after intentionally reducing plasma cholesterol returned this parameter to normal levels and cytoplasmic CE droplets had been cleared (Jerome and Lewis, 1990). The loss of lysosomal hydrolysis was also demonstrated in THP-1 MOs exposed to mildly oxLDL or aggregated LDL (aggLDL). Initially, CE hydrolysis within lysosomes and FC efflux from this organelle was not inhibited. After prolonged exposure to these lipoproteins (>48 h), however, CE hydrolysis became increasingly inhibited and lysosomes began to accumulate CE (Yancey and Jerome, 2001; Griffin et al., 2005). A further study suggested the inhibition of lysosomal function is a general effect not related to the oxidation status of a given lipid. Here, the loss of lysosome function, including a reduction in LAL-dependent CE hydrolysis over time, was verified in MOs loaded with either mildly oxLDL or aggLDL or cholesteryl ester-rich lipid dispersions (Cox et al., 2007).

Furthermore, lysosomal dysfunction in oxLDL-loaded MOs is irreversible (Jerome et al., 2008). Collectively, these data indicate that inhibition of LAL activity, as well as of other lysosomal enzymes such as cathepsins (O'Neil et al., 2003), are the result of permanent alterations in lysosome function following excess lipid loading. The net effect is an accumulation of lysosome cargo (Figure 1). These findings are in agreement with data produced in our group using cholesteryl hemisuccinate (Chems), a member of the cholesteryl hemiester family. Cholesteryl hemiesters are produced as result of CE oxidation and have been detected in human atheromata (Hutchins et al., 2011) and oxLDL (Kamido et al., 1995). MOs exposed to LDL enriched in Chems demonstrated an irreversible accumulation of undigested lipid in enlarged lysosomes, which had increased Chems content in lysosomal membranes (Estronca et al., 2012; Domingues et al., 2017).

### Functional impairment of lysosomes stimulates inflammasome assembly and pro-inflammatory cytokine release

Lysosome function in both health and disease is intrinsically linked to cytokine release. Cytokines are synthesized by MOs after cell activation and secreted via the constitutive (or continuous) secretory pathway or by non-conventional secretion. The majority of cytokines expressed in MOs are processed and transported through the constitutive pathway; however, some require non-conventional secretion for cellular release. Secretory lysosomes facilitate this secretion and are also able to degrade inflammatory cytokines to regulate the immune response to an external stimulus, such as lipopolysaccharide (LPS) and adenosine triphosphate (ATP) (Murray and Stow, 2014). Lysosomes modulate cytokine production in other fundamental ways. For instance, TMEM9B, a glycosylated protein localized in lysosomal membranes regulates Tumour necrosis factor (TNF-) induced Interleukin-6 (IL-6) and IL-8 mRNA expression. It is also necessary for TNF-, IL-6 and Toll-like receptor 2- (TLR2-), TLR3-, and TLR4- induced IL-8 expression, demonstrating its control of pro-inflammatory signaling cascades (Dodeller et al., 2008). Additionally, lysosomes can down-regulate TLR9-mediated proinflammatory cytokine and type I IFN production in MOs by degrading the receptor following its translocation from the ER (Yao et al., 2009).

**Figure 2:**
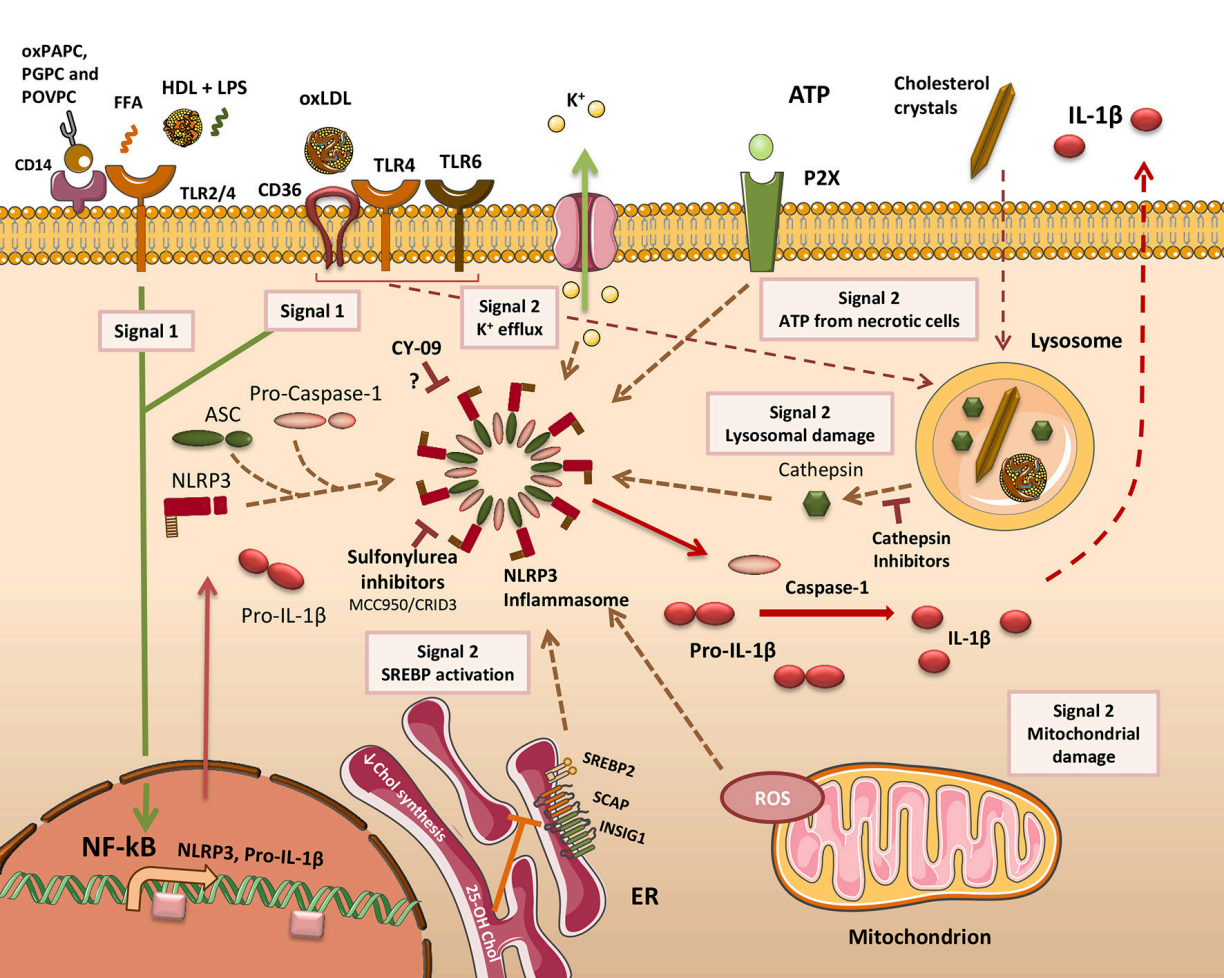
Mechanisms of inflammasome activation in atherosclerosis. To facilitate the release of IL-1β (& IL-18) in macrophages (MO), two distinct signals must be delivered. Firstly, cells need to be primed (Signal 1) to activate the NF-κB-responsive genes *NLRP3* and *IL-1*β. In atherosclerosis, a broad range of lipid and lipoprotein agonists provides this signal by activating cell surface pattern recognition receptors (PRRs). 1-palmitoyl-2-arachidonoyl-sn-3-glycero-phosphorylcholine (oxPAPC), 1-palmitoyl-2-glutaroyl-sn-glycero-phosphatidylcholine (PGPC), and 1-palmitoyl-2-(5-oxovaleroyl)-sn-glycero-phosphatidylcholine (POVPC) prime cells through CD14. Toll-like receptor 2 (TLR2) has also been described as an oxPAPC receptor. Free fatty acids (FFA) prime MOs through a TLR2-TLR4 signaling complex. The ApoAI moiety in High-density lipoprotein (HDL) can co-activate peritoneal MOs along with Lipopolysaccharide (LPS) through TLR2 or TLR4. Oxidized LDL (oxLDL) can prime MOs via signaling through a CD36-TLR4-TLR6 receptor complex. CD36 can additionally promote oxLDL uptake and its conversion into cholesterol crystals within phagolysosomes. The second signal (Signal 2) is required for assembly of the canonical NACHT, LRR and PYD domains-containing protein 3 (NLRP3) inflammasome complex. A number of different activating stimuli are able to deliver this signal. These include increased sterol-regulatory element-binding protein 2 (SREBP2) activity, mitochondria-derived reactive oxygen species (ROS), lysosome damage leading to cathepsin release, and exogenously-derived ATP from adjacent necrotic cells. Activation of surface P2X receptors leads to K^+^ efflux through ion channels. Inflammasome activation produces mature caspase-1 which cleaves pro-IL-1β (or pro-IL-18), forming mature bioactive IL-1β that is released from the cell. Cathepsin inhibitors diminish IL-1β release by inhibiting NLRP3 inflammasome activation. Other chemicals shown to inhibit the NLRP3 inflammasome include MCC950, which also reduces atherosclerosis in mice, and CY-09 that works through an unknown mechanism. SCAP, SREBP cleavage-activating protein; INSIG, insulin-induced gene protein.

IL-1β has a fundamental role in establishing and driving the pathogenesis of atherosclerosis. It stimulates Mos and MOs as well as endothelial cells (EC) and smooth muscle cells (SMC) to secrete proinflammatory cytokines and chemokines (Libby, 2017). These cells also release increased amounts of specific matrix metalloproteinases (MMPs) in response to IL-1. These include MMPs with defined roles in EC erosion, SMC proliferation, remodeling and migration, and MO-mediated plaque rupture. IL-1 induced chemokines attract phagocytes to a plaque. Effects on cardiomyocyte function are also known (Libby, 2017). The significance of its role in atherosclerosis formed the basis of the CANTOS clinical trial (Ridker et al., 2017), which is discussed in a subsequent section within this article entitled Therapeutic Interventions to Treat Atherosclerosis. Given this critical role, here we will discuss the impact of lysosome function and dysfunction on its release. Five potential mechanisms of IL-1β release have been described (Eder, 2009; Martín-Sánchez et al., 2016), one of which is the exocytosis of secretory lysosomes (Figure 1). Early evidence of this process came from Mos in which the lysosomal membrane marker LAMP-1 co-localized with intracellular pro-IL-1β, pro-caspase-1 and cathepsin D. The latter was subsequently detected extracellularly (Andrei et al., 1999). In MOs, but not Mos, two distinct signals are required for IL-1β release. The first of these is a “priming” signal (Signal 1), which leads to synthesis of a biologically inactive precursor form of pro-IL-1β. Examples of lipid ligands that prime MOs are discussed in the section entitled A Broad Spectrum of Lipid and Lipoprotein Species Modulate Inflammation in Macrophages. To produce a mature, bioactive form of IL-1β, it is necessary for the pro-peptide to be cleaved by caspase-1 (Black et al., 1988). To synthesize a mature form of caspase-1, a multiprotein inflammasome complex needs to be activated and formed in the cytosol. Inflammasome assembly requires a second signal (Signal 2), which can be delivered by a number of different sources, including dysfunctional lysosomes (Tall and Yvan-Charvet, 2015) (Figure 2). Differences between the two cell types are believed to reflect an adaptation by monocytes to facilitate their role in patrolling a typically pathogen-free environment, hence a requirement to rapidly respond to danger signals. Macrophages, by contrast, are tissue resident so are almost continually exposed to a range of foreign antigens. Being overly sensitive to their presence would be dangerous for the host (Van De Veerdonk et al., 2011). Sterol-loaded lysosomal membranes have increased permeability, causing their contents to leak into the cytosol. This has been shown in MOs that demonstrated a leakage of lysosomal enzymes after cells had been incubated with oxLDL, a mixture of cholesterol oxidation products or cholesterol crystals (Li et al., 1998; Yuan et al., 2000; Emanuel et al., 2014). OxLDL entry into MOs led to the formation of cholesterol crystals after only 1 h (Duewell et al., 2010). These crystals grew in magnitude over time, were deposited in phagolysosomes and ruptured lysosomal membranes. Both oxLDL and exogenously applied cholesterol crystals induced Il-1β release in the absence of other stimuli, demonstrating they provide both signals (1 and 2) required to release this cytokine in MOs. In cathepsin B and L knockout (KO) bone marrow derived MOs (BMDM), crystal-stimulated Il-1β release is diminished but not abolished, confirming these cysteine proteases derived from dysfunctional lysosomes provide some of signal 2 (Duewell et al., 2010). The precise mechanism of cathepsin-mediated NACHT, LRR and PYD domains-containing protein 3 (NLRP3) inflammasome assembly remains elusive. A potential consequence of leaky lysosomes is an increase in the excretion of lysosomal enzymes, which may explain the presence of extracellular LAL in atherosclerotic lesions (Tapper and Sundler, 1992; Hakala et al., 2003).

In addition to the unregulated leaking of lysosomal proteases, particulate matter also triggers K^+^ efflux from the cell (Figure 2). This is essential for the activation of NLRP3 inflammasomes induced by lysosomal destabilization (Muñoz-Planillo et al., 2013; He Y. et al., 2016). The mechanism linking particulate matter-induced lysosomal rupture to K^+^ efflux is undetermined at present. Calcium phosphate also exists as particulate matter, and has been shown to accumulate in atherosclerotic lesions (Hirsch et al., 1990). Calcium phosphate crystals also initiate NLRP3 inflammasome assembly through lysosomal membrane rupture and cathepsin B release. They facilitate the release of both IL-1β and IL-1α in MOs (Usui et al., 2012). Data produced in our group observed that in MOs incubated with Chems, lysosomes become dysfunctional, and cells become overtly proinflammatory, releasing IL-1β in addition to other inflammatory cytokines (Domingues et al., 2017). However, in contrast to oxLDL and cholesterol crystals, Chems does not deliver both of the signals required for IL-1β release, with exposure to a low dose of LPS also necessary. In this experiment, a non-toxic concentration of Chems was used and it may be possible that with higher concentrations, lysosomes become more damaged, providing the second signal for IL-1β release.

### ER stress and mitochondrial dysfunction occur in response to lipid loading

Under homeostatic conditions, the ER regulates the synthesis, processing, and folding of secretory proteins. An imbalance occurs when the synthesis of proteins overwhelms the folding capacity of the ER, leading to the accumulation of misfolded and unfolded proteins in the lumen. This is defined as ER stress and is implicated in the pathogenesis of many inflammatory disorders. Under ER stress conditions, a compensatory system called the unfolded protein response (UPR) is mobilized to mitigate ER stress signaling to restore homeostasis. Under pathologically chronic ER stress, cell dysfunction and disease manifest, potentially leading to cell death. Three upstream ER stress transmembrane sensor proteins respond to the presence of misfolded and unfolded proteins by triggering the UPR. They are activating transcription factor 6 (ATF6), inositol-requiring enzyme 1α (IRE1α), and protein kinase R-like endoplasmic reticulum kinase (PERK). The latter augments the translation of ATF4, inducing the production of C/EBP-homologous protein (CHOP), which instigates a number of restorative functions to temper transient ER stress (Tabas, 2010). One of the now well-established consequences of ER stress is activation of the NLRP3 inflammasome (Menu et al., 2012). The precise molecular mechanism underlying this pathway is poorly understood, though it may be UPR independent (Menu et al., 2012). Regardless of the mechanism, ER stress is evidently a critical factor in the progression of atherosclerosis and has been reviewed extensively (Hotamisligil, 2010; Tabas, 2010). Through determining CHOP expression (amongst other markers) in MOs from early fatty streaks and advanced lesions, ER stress was manifestly elevated during the progression of atherosclerosis in chow- or Western diet-fed ApoE^−/−^ mice. CHOP expression levels were found to increase with disease severity (Feng et al., 2003; Zhou et al., 2005). Similar findings were subsequently revealed in humans with unstable plaques (Myoishi et al., 2007). ER stress activates SREBP2, which delivers a source of “Signal 2” to activate assembly of the NLRP3 inflammasome (Reboldi et al., 2014) (Figure 2).

Mitochondrial dysfunction is also prominent during atheroprogression. Amongst the multitude of processes that occur within mitochondria, oxidative phosphorylation, through the production of ATP, liberates moderate (physiological) levels of superoxide, the bulk of which are converted to hydrogen peroxide by superoxide dismutase. This process remains homeostatic under normal conditions, however, under pathophysiological conditions; chronic overproduction of ROS arises from excessive mitochondrial oxidative stress. The link between mitochondrial oxidative stress (MitoOS)-induced pathology and atherosclerosis was made in an elegant study using transgenic mice in which MitoOS had been suppressed in lesional MOs. These mice exhibited a reduction in lesional area of the aortic root, a lower abundance of Mos in these lesions and a concomitant reduction in Ccl2 and RelA phosphorylation, indicating the NF-κB signaling pathway responsible for proinflammatory cytokine production was also downregulated. Mitochondrial damage or dysfunction delivers, through the production of ROS, another source of “signal 2” to initiate inflammasome assembly (Figure 2).

Uncoupling oxidative phosphorylation in mitochondria reduces ATP production and may also decrease ROS production in a cell. Despite this, a study looking at the effects of oleic acid in mice found that this unsaturated fatty acid induced mitochondrial respiratory uncoupling and inflammasome independent IL-1α release to drive atherogenesis (Freigang et al., 2013). Il-1α release was mediated by an increase in calcium efflux from the mitochondria to process inactive pro-IL-1α via calcium-dependent calpains. Intriguingly, this study described mitochondrial dysfunction in the absence of overt ROS-mediated IL-1β production with IL-1α the more prominent cytokine (Freigang et al., 2013). This suggests the considerable effort undertaken to decipher the mechanisms of IL-1β production in atherosclerotic MOs may only tell part of the story.

Some of the research into these organelles is now aiming to identify specific dietary factors that promote ER stress, mitochondrial oxidative stress and/or ROS overproduction. For example, ROS-mediated NLRP3 inflammasome activation was established in BMDMs exposed to long-chain saturated fatty acids (SFA) (Wen et al., 2011). More recently, SFAs were shown to induce an ER stress response via an inositol-requiring enzyme 1-α (IRE1-α)-dependent pathway in BMDMs, promoting NLRP3 activation and IL-1β release (Robblee et al., 2016), though the long chain SFA palmitate does not induce these changes by directly binding TLR4 (Lancaster et al., 2018). An assessment of additional ligands that may affect these organelles is likely to be carried out in the near future.

### Apoptosis and inefficient efferocytosis contribute to plaque progression

In advanced atherosclerotic lesions, MO apoptosis, often caused by ER stress and lysosome dysfunction; and plaque necrosis are prominent features of the disease pathology (Subramanian et al., 2015; Tabas and Bornfeldt, 2016). Both caspase-1-dependent pyroptosis (Lin et al., 2013b) and receptor-interacting protein kinase 3 (RIP3)-dependent necroptosis (Lin et al., 2013a) are additional forms of lipid-mediated cell death to have been observed *in vitro*, and are likely to also contribute to lesional MO death *in vivo*.

Necrotic cores arise following the death of advanced lesional MOs and within these lipid-rich regions, another example of cell dysfunction occurs to exacerbate plaque stability. The major functional feature of MOs is phagocytosis, which under infectious conditions involves degrading pathogen-derived antigens, which become presented on their surface by MHC molecules. In atherosclerotic plaques, MOs perform a related yet distinct function known as efferocytosis. It involves removing apoptotic and other dying MOs by engulfment to prevent the establishment of a necrotic core, resolving inflammation. Generally, efferocytic MOs are more resistant to lipid accumulation, have an alternative anti-inflammatory phenotype, and reside in more stable regions of a plaque (Chinetti-Gbaguidi et al., 2015). In advanced plaques, efferocytosis becomes defective, leading to an accumulation of apoptotic MOs contributing to plaque necrosis and increased inflammation (Tabas, 2010).

## The inflammatory status of lesional macrophages is regulated at multiple levels

MO-driven inflammation is fundamental to the progression of atherosclerosis, but precisely how these cells become polarized and evolve is an open debate. Data acquired over the past decade and a half has revealed a considerable amount of MO heterogeneity exists within atherosclerotic plaques (Chinetti-Gbaguidi et al., 2015). Depending on the lesional stimulus encountered, Mos differentiate toward a particular phenotype, which is characterized by cytokine release, surface markers, lipid or iron handling capabilities, and functions such as phagocytic capacity (Colin et al., 2014). MOs exhibit considerable plasticity and are thought to be able to switch their phenotype and inflammatory status in a plaque upon exposure to alternative stimuli (Chinetti-Gbaguidi et al., 2015). It is important to remember these phenotypes have been defined *in vitro*, under simplified conditions, and usually in response to a single stimulus. Given the complexity of the lesional microenvironment, they are unlikely to exist in their purest form in plaques and instead represent snapshots of a whole spectrum of states (Tabas and Bornfeldt, 2016). This viewpoint is gathering momentum (Nahrendorf and Swirski, 2016; Tabas and Bornfeldt, 2016), and mirrors a similar move by the field to redefine MO nomenclature according to functional status (Mosser and Edwards, 2008; Martinez and Gordon, 2014; Murray et al., 2014). So a more realistic view of plaque MO phenotype is a fluid, continually evolving entity, with the inflammatory status determined by a balance between the multiple signals they receive *in vivo*, lipid uptake and cellular metabolism of fatty acids (Tabas and Bornfeldt, 2016). Historically, a combination of technical limitations and a generally reductive experimental approach have failed to adequately evaluate plaque MO heterogeneity *in vivo*. Using a pre-defined panel of markers or secreted molecules such as cytokines, usually chosen to fit with the data from *in vitro* studies, is inherently biased and has undoubtedly limited the discovery of novel phenotypes. This has led to interpreting *in vivo* function by analogy. High-resolution data on other cell types known to reside in lesions has also been lacking. A brand new study has used single cell RNA sequencing to characterize aortic MOs and dendritic cells (DCs), revealing hitherto unknown levels of diversity amongst these cell types and their distinct subsets *in vivo* (Cochain et al., 2018). Alternative future approaches may include mass cytometry (Spitzer and Nolan, 2016) and ribosome profiling (Brar and Weissman, 2015), however, the latter has yet to be developed at the single cell level. Coupling this with a thorough metabolic and epigenetic characterization would greatly advance our understanding of how these cells become dysregulated under disease conditions.

### Macrophage immunometabolism in response to lipids

Our understanding of what constitutes inflammation and how it is controlled is also changing (Editorial, 2017). Inflammation and metabolism are interminably entwined with both inflammatory and metabolic phenotypes capable of regulating one another. It is now well-known that MO phenotype and function are controlled by mitochondria acting as the hub for intrinsic metabolic pathways (O'Neill and Pearce, 2016). Glycolysis promotes pro-inflammatory functions in LPS + IFNγ-activated MOs, whilst in alternatively, IL-4 polarized MOs, oxidative phosphorylation regulates their anti-inflammatory phenotype (O'Neill and Pearce, 2016). This picture has become a little blurred more recently with both metabolic processes now recognized as having dichotomous roles in driving these opposing strands of inflammation (Van Den Bossche et al., 2017). A mechanistic understanding of how other ligands drive immunometabolism in MOs is currently lacking as most of the data accrued thus far is from *in vitro* polarized cells. A handful of studies have begun to assess the impact of lipid species on MO metabolism and inflammation in atherosclerosis (Bories and Leitinger, 2017). For instance, oxLDL-stimulated MOs are pro-inflammatory and exhibit enhanced glycolysis (Tawakol et al., 2015). This study also identified hypoxia-inducible factor 1-α (HIF1α) as the key driver of glucose uptake and the expression of glycolytic enzymes HK2 and 6- phosphofructo-2-kinase/fructose-2,6-biphosphatase 3 (PFKFB3). Another study also found that glutamine facilitates the lipotoxic effects of dietary fatty acids in MOs. In its absence, a reduction in lysosome dysfunction, inflammasome assembly and cell death was observed (Wen et al., 2011; He L. et al., 2016). In IL-4 polarized MOs, fatty acid oxidation following exogenous lipid uptake and lysis are important for the engagement of elevated oxidative phosphorylation and to preserve their anti-inflammatory phenotype (Huang et al., 2014). Most of this data, however, has been derived *in vitro*, so the immunometabolic status of plaque MOs and how this evolves within the dynamic lesional microenvironment is mostly unknown. Consequently, the impact it may have on the progression of atherosclerosis remains scant.

### Dysregulated inflammation in foam cells

Lesional MO abundance correlates with the progression and stability of plaques. The chronic inflammatory state within a plaque has a significant impact on this, as lipid-loaded foam cells are known to release inflammatory cytokines (Moore et al., 2013) and may also undergo necrosis to propagate the disease. The progression of inflammation in cells that are already full of lipid has therefore received some attention. A study on this theme (Spann et al., 2012) revealed that in peritoneal foam cells removed from LDLR KO mice on a high cholesterol, high fat diet; a significant number of pro-inflammatory genes had down-regulated expression. Most of these genes were highly expressed in response to a Tlr4 agonist in a previous study referenced by the authors. Amongst those demonstrating the highest degree of transcriptional suppression were Il-1β, Cxcl9, and Cxcl10. These findings were consolidated *in vitro* using murine peritoneal MOs (pMOs) and human Mo-derived MOs. In both cell types, cholesterol loading followed by Tlr4 stimulation [with Kdo(2)-lipid A, KLA, a TLR4-specific agonist] led to a significant inhibition of Cxcl9 and Cxcl10 expression, compared with KLA-treated cells (Spann et al., 2012). A similar trend was observed in elicited pMOs loaded with acetylated-LDL (Suzuki et al., 2012). In this study, a panel of LPS-inducible pro-inflammatory genes had down-regulated expression when subsequently challenged with this agonist, compared with cells that were only exposed to LPS (Suzuki et al., 2012). In human M-CSF-differentiated MOs converted into foam cells with acetylated LDL, a comparable picture emerged. Foam cells, along with untreated MOs, were exposed to polarizing stimuli (LPS & IFNγ) after which, the expression of several key pro-inflammatory cytokines was quantified. For every gene examined, lipid loading led to lower relative expression levels (Da Silva et al., 2016). A recent study attempted to decipher the mechanism underlying this trend. In line with previous reports, a general blunting of the pro-inflammatory response in lipid-loaded MOs was observed (Jongstra-Bilen et al., 2017). Specifically, exposure to oxLDL inhibited pro-inflammatory cytokine expression in pMOs subsequently stimulated with Tlr2, Tlr3, Tlr4, or Tlr9 agonists (Jongstra-Bilen et al., 2017). Mechanistically, the NF-kB RelA/p65 subunit demonstrated reduced binding to Il-6 and Ccl5 promoters, which was partially reversed with a broad-spectrum histone deacetylase inhibitor (Jongstra-Bilen et al., 2017). These results confirm that oxLDL can instigate epigenetic changes that regulate inflammation, corroborating previous in depth studies (Chen et al., 2011; Reschen et al., 2015). Further studies could seek to better characterize these foam cells and also identify whether different lipid species can dampen a common cohort of lipid handling and proinflammatory genes. In assessing why peritoneal foam cells had inhibited pro-inflammatory cytokine expression, (Spann et al., 2012) speculated that exogenous stimuli from within the artery wall might elicit a pro-inflammatory state in lesional MOs. Regardless, the suppressive effect of lipid loading on inflammation is clear though may be context-dependant with cell origin, the limitations of *in vitro* conditions, and technical differences in oxLDL preparation potentially affecting outcomes.

### Senescent plaque macrophages are dysfunctional

Despite the very clear and reproducible effect described above, numerous studies over many years have reported that lipid-laden plaque MOs can be markedly pro-inflammatory in progressing plaques. Recent research has elegantly demonstrated a link between their inflammatory capacity, advanced plaque stability and cell senescence (Childs et al., 2016). The study revealed that pro-inflammatory foamy MOs rapidly emerge and colonize fatty streak lesions in the inner curvature of the aortic arch in LDLR KO mice on a high fat diet. After only 9 days, some of these cells expressed senescence markers and by selectively eliminating them, lesional pro-inflammatory marker expression and streak size was reduced (Childs et al., 2016). In established plaques, removing senescent cells also diminished pro-inflammatory marker expression, reduced the size and amount of lesions and halted disease progression. Senescent cells isolated and flow sorted from advanced lesions expressed far higher levels of pro-inflammatory markers when compared with non-senescent cells (Childs et al., 2016). This study adds another layer of complexity to our understanding of inflammation in atherosclerotic MOs. It does, though, carry a degree of controversy in the context of other recent studies. For instance, plaque inflammation is greatly enhanced by proliferating MOs that drive and increase the severity of atherosclerosis (Tang et al., 2015). By contrast, senescent cells do not divide but they are also inflammatory and drive atherosclerosis (Childs et al., 2016) in the absence of proliferation, which was not quantified. It is possible that smooth muscle cell-like MO infiltration may be involved and contribute to perpetuating inflammation in the senescent cell-rich plaque areas.

### Circadian rhythms regulate inflammatory function

An important concept under an increasing amount of scrutiny is the circadian control of inflammation. A landmark study on the link between the circadian clock and immune function revealed that the rhythmic control of cytokine release in MOs is profound. The release of Il-6 & Tnfα in LPS-stimulated *ex vivo* murine MOs was shown to oscillate, confirming its regulation is cell intrinsic, not systemic. Furthermore, multiple genes at all cellular stages (surface, cytoplasmic, nuclear) of LPS-induced cytokine expression were evidently under circadian control (Keller et al., 2009). Recent examples of circadian rhythms pertaining to key processes that underpin atherogenesis and related vascular diseases have emphasized its importance. Individuals that suffer an acute myocardial infarction early in the morning experience greater damage to the heart and have a poorer recovery and long-term prognosis. This is attributable to a circadian controlled fluctuation in neutrophil recruitment to the heart, which is governed by oscillations in their surface expression of CXCR2 (Schloss et al., 2016). This study refers only to the circadian control of immune function affecting cardiac events. Yet, the strong connection between atherosclerosis and vascular disease opens up the possibility that the circadian control of inflammatory lesional components may also contribute to the timing and severity of an event, along with the known morning blood pressure surge (Marfella et al., 2007). In support of this, many fundamental components driving atherogenesis, plaque progression and plaque stability are known to be under circadian control. These include the expression of intercellular adhesion molecule 1 (ICAM-1) and several chemokines in endothelial cells; metalloproteinase expression in VSMCs; and cytokine, chemokine, TLR, and lipid handling genes in MOs (Mcalpine and Swirski, 2016). Phagocytosis, an indispensable function performed by MOs, has also recently been shown to be under circadian control (A-Gonzalez et al., 2017). Phagocytic MOs were shown to be anti-inflammatory, had dampened Il-1β expression regardless of the tissue source, and maintained tissue homeostasis by removing dead or dying cells–a process called efferocytosis (which has been reviewed extensively elsewhere, Tabas, 2010). This limited inflammatory cytokine production, thereby reducing leukocyte migration (A-Gonzalez et al., 2017). Whilst the relevance of this study was not discussed in the context of atherosclerosis, the function of efferocytic plaque MOs is conspicuously similar to the data they acquired from phagocytic MOs in other tissues, further emphasizing the probable circadian influence on the disease.

## A broad spectrum of lipid and lipoprotein species modulate inflammation in macrophages

The biological properties of lipids and lipoproteins have been studied for decades. A major focus of this research has been to decipher their inflammatory capacity and, consequently, the impact they may have on atherosclerosis and other chronic diseases. Given the increasing desire for more translational studies and improved therapies, efforts to comprehend the fundamental elements of cell and organelle dysfunction underlying inflammatory changes are underway. One of the most important concepts to have recently emerged in the field of immunology is that of innate immune memory or “trained immunity.” This arm of the immune system has historically been viewed as non-specific and lacking the capacity for memory. Trained immunity dictates that the innate immune response has the ability to adapt (Netea and Van Der Meer, 2017). Given the long-term chronic inflammation that characterizes atherosclerosis and the initial priming signals mediated by lipids, there was inevitably an interest in evaluating the effect in this context. So, do lipid signals train cells of the innate immune response? It appears that they do as exposure to atherogenic mediators induces a long-term activated phenotype in innate immune cells. Mechanistically, there are quantifiable changes in epigenetic programming and immunometabolism that define this altered phenotype, which modulates the perpetual inflammatory environment within a plaque. These findings are in contrast with those discussed in the subsection entitled “Dysregulated Inflammation in Foam Cells,” and this may be related to the extent (and type) of lipid loading in the cells examined. Dietary factors are also known to affect the trained phenotype (Christ et al., 2016). Therefore, a better characterization of well-known and more recently discovered lipid mediators of inflammation and precisely how they alter cell function is essential. This is relevant for “priming” signals that rapidly affect transcription and intracellular signals; and also for metabolic, epigenetic, and inflammatory effects that occur following lipid ingestion and processing. This section will discuss how a selection of these lipids and lipoproteins affect inflammation and the potential consequences for cell physiology.

Lipids and lipoproteins undergo considerable modification following oxidative damage, rendering them profoundly immunogenic. Oxidized host molecules have an altered appearance or conformation due to the exposure of distinct moieties known as oxidation-specific epitopes (OSEs) (Miller et al., 2011). These OSEs operate as *de facto* damage-associated molecular patterns (DAMPs), and are therefore recognized by host pattern recognition receptors (PRRs), similar to how bacterial pattern-associated molecular patterns (PAMPs) are recognized by TLRs (Miller et al., 2011). Despite this, these DAMPs do not faithfully recapitulate the effects of microbial PAMPs. They are recognized, for instance, by TLRs & scavenger receptors, on host cells, inducing pro-inflammatory cytokine production. However, the precise nature of these interactions differs from pathogen recognition (Miller and Shyy, 2017). When lipids are oxidized to the extent that they become fragmented, producing “end products” of lipid oxidation, they are consistently pro-inflammatory. Some of the best-known examples are reviewed in detail elsewhere (Miller and Shyy, 2017).

### Oxidized low-density lipoprotein and cholesterol crystals

To better understand lipid-mediated sterile immune responses *in vivo*, many researchers have established *in vitro* models using oxLDL, given its prevalence in atherosclerotic patients. Similar to “glaucoma,” oxLDL is an umbrella term that describes a vast mixture of over 3000 molecules; comprised of apolipoprotein B-100, cholesteryl esters, free cholsterol, phospholipids, and triglycerides (Levitan et al., 2010). Although oxLDL is usually reported as being pro-inflammatory, particularly in Mos and MOs (Stewart et al., 2010; Sheedy et al., 2013; Tiwari et al., 2014; Rhoads et al., 2017), there are context-dependent examples of where it is not, as discussed in the previous section. As well as the cell types used, alternative outcomes may also be a consequence of the different methods used to modify LDL *in vitro*. It is probable that across the many studies published, no two batches of oxLDL are exactly alike, with variable amounts of oxidized immunogenic components affecting the intensity of inflammatory responses in MOs. The study describing how oxLDL promotes inflammation (Duewell et al., 2010) is discussed in the section entitled Atherosclerosis Involves Multiple Examples of Dysregulated Cell Function. Another key concept established by this study was the activation of NLRP3 inflammasome assembly by oxLDL & cholesterol crystals for both Il-1β release *in vitro* and atherogenesis *in vivo*. The mechanism of Nlrp3 activation was elucidated further in mouse MOs *in vitro*. OxLDL uptake, formation of cholesterol crystals and subsequent inflammasome priming and Il-1β release were dependent on Cd36 (Sheedy et al., 2013). In peritoneal MOs, Nlrp3 priming also required Tlr4 and Tlr6 and was driven by OxLDL-induced ROS production (Sheedy et al., 2013). Subsequent studies have reinforced the importance of the NLRP3 inflammasome as a critical driver of the disease (Paramel Varghese et al., 2016; Fuster et al., 2017), and therefore a key target for therapeutic intervention (Sheridan, 2017). Indeed, selectively inhibiting Nlrp3 restricts oxLDL uptake by THP-1 MOs, thus impairing foam cell formation. This inhibition also led to the downregulation of Cd36, which, in the context of the findings by Sheedy et al. (2013), almost certainly limited the appearance of foam cells (Chen et al., 2018). Several recent reviews have also focussed on NLRP3 as nexus between oxidized lipids and inflammatory cytokine release in atherosclerosis (Baldrighi et al., 2017; Patel et al., 2017; Hoseini et al., 2018). In contrast to the majority of available literature, one study found that in ApoE^−/−^ Nlrp3^−/−^ C57/BL6 mice on a high fat diet there was no difference in the degree of atherosclerosis between these and wild-type controls after 11 weeks. This suggested that the NLRP3 inflammasome had no impact on atherogenesis (Menu et al., 2011). This outcome has been directly contradicted in a recent study using a highly specific NLRP3 inflammasome inhibitor (MCC950) (Van Der Heijden et al., 2017). Here the data showed that atherosclerosis in ApoE^−/−^ mice receiving MCC950 is reduced after 4 weeks of lesional development compared with controls. These mice were fed a Western diet containing cholesterol and butter. There are tangible differences between the methods used in these two studies, which likely affected the outcome. MCC950-mediated NLRP3 inhibition has already been shown to be highly specific *in vivo* and in *ex-vivo* murine Mos and MOs (Coll et al., 2015; Primiano et al., 2016), though the mechanism of its action remains elusive. A recent study has described another specific NLRP3 inhibitor (CY-09). This compound targets the inflammasome by inhibiting NLRP3 oligomerization and ASC recruitment and may offer a safe and selective therapeutic benefit to patients with atherosclerosis in future (Jiang et al., 2017). The use of cholesterol crystals was central to defining the role of NLRP3 in atherosclerosis (Duewell et al., 2010) and dietary cholesterol has also been shown to induce intestinal inflammation *in vivo* in zebrafish (Progatzky et al., 2014). The dietary form seems to lack the potency of crystals, as it requires a priming signal delivered by commensal microbiota to induce NLRP3-dependent inflammation (Progatzky et al., 2014).

### High density lipoprotein

Whilst LDL and its oxidized derivatives are typically pro-inflammatory, high-density lipoprotein (HDL) is commonly perceived as being anti-inflammatory, with a wealth of research supporting this view (Rye and Barter, 2014). HDL has a range of prominent functions, one of which is reverse cholesterol transport (Heinecke, 2012). Two membrane ATP-binding cassette transporters, ABCA1 and ABCG1, upregulated in cholesterol-rich MOs are responsible for mobilizing HDL to the liver via the circulation. The importance of HDL in limiting inflammation has been demonstrated in mice that have these transporters deleted in their MOs (ABC^DKO^ mice), but not in their stem and progenitor cell populations. In LDLR^−/−^ mice transplanted with ABC^DKO^ bone marrow (BM), both inflammation and atherosclerosis are increased (Westerterp et al., 2013). In plaque MOs lacking these transporters, pro-inflammatory cytokine (*Il-1, Il-6*) and chemokine [*Ccl3* (*Mip1*α), *Ccl2 (Mcp1)*] expression is significantly elevated (Westerterp et al., 2013). Directly challenging cells with HDL is an alternative approach that has been used to study its impact on inflammation. In MOs loaded with acetylated LDL, subsequently pre-treated with HDL then stimulated with LPS, a significant inhibition of LPS-mediated gene expression was observed (Suzuki et al., 2010). HDL selectively inhibited anti-viral response genes from the type I IFN response pathway that were TRAM/TRIF-dependent. Some of these genes, such as *Nos2* and *Cxcl10*, have described roles in promoting inflammation. Notably, pre-treatment with HDL did not inhibit MyD88-dependent pathway genes, which included the pro-inflammatory cytokines *Il-1b, Il-6*, and *Tnfa* (Suzuki et al., 2010). A further study characterized the molecular mechanism underlying the HDL-driven suppression of inflammation (De Nardo et al., 2014). Two forms of HDL were used to similar effect—native HDL (nHDL) isolated from human plasma donors, and reconstituted HDL [HDL; pure human apolipoprotein A-I (ApoA-I) with phospholipids]. In mice injected with nHDL and subsequently injected with the Tlr9 agonist CpG-DNA, there were significantly lower levels of serum Il-6 and Il-12p40 1 h later. The identical result was observed with HDL, as well as lower levels of Tnf and Ccl2, but also Il-10. These data were validated *in vitro* in BMDMs pre-treated with HDL and subsequently challenged with a range of Tlr agonists. Mechanistically, HDL was shown to drive the expression of the transcriptional repressor, activating transcription factor 3 (ATF3), to downregulate pro-inflammatory cytokine production (De Nardo et al., 2014).

Two prominent studies have presented conflicting data, both convincingly showing HDL is pro-inflammatory in mouse MOs. The first of these (Smoak et al., 2010) used Apo-AI (the major protein constituent of HDL) to stimulate pMOs from Tlr2, Tlr4, MyD88, Tirap, and Trif knockout mice alongside wild type controls. All of these receptors and signaling adaptors respond to Apo-AI. MyD88^−/−^ pMOs were also used to show ApoA-I induces release of Il-1α, Il-1β, Il-6, Tnf-α, Cxcl1, Cxcl2, and Ccl2 (De Nardo et al., 2014). The second, very recent assessment of HDL revealed that its pro-inflammatory capacity is cell-type-dependant (Van Der Vorst et al., 2017). In endothelial cells and smooth muscle cells, the same group previously demonstrated that HDL is anti-inflammatory (Bursill et al., 2010; Van Der Vorst et al., 2013). Yet, they showed it is evidently pro-inflammatory in mouse MOs. HDL in isolation induced dose-dependent increases in Tnfα and Il-12 transcription, but the opposite trend was observed for Il-10. BMDMs stimulated with a range of nHDL concentrations then a low fixed [LPS] dose-dependently released Tnfα & Il-12. Intriguingly, BMDMs stimulated with a fixed concentration of either nHDL, reconstituted HDL (rHDL; ApoA-I complexed with 1-palmitoyl-2-linoleoyl-PC) or commercially purchased HDL (cHDL) then a low fixed [LPS] released Tnfα & Il-12 but failed to do so without the LPS present, suggesting HDL and LPS synergistically regulate cytokine release (Van Der Vorst et al., 2017). These findings were broadly replicated *in vivo*. In line with this finding, minimally modified LDL (mmLDL) and a low dose of LPS synergistically increase chemokine release in MO cell lines (Wiesner et al., 2010). Some of the experiments carried out by De Nardo et al, were replicated using soybean phosphatidylcholine rHDL, but got contrasting data (Van Der Vorst et al., 2017). They saw a similar reduction in Il-12 and IL-10 release, but Tnfα release and Atf3 expression were unaffected. They also speculated that the anti-inflammatory function of the rHDL used in the De Nardo study was caused by the soybean content of the preparation (Van Der Vorst et al., 2017). Regardless, it is clear that understanding the biology of HDL remains a work in progress, with both pro- and anti-inflammatory functionality and its capacity to be dysfunctional identified and reviewed many years ago (Navab et al., 2009).

### Phospholipids

Oxidized phospholipids (oxPLs), formed following polyunsaturated fatty acid oxidation, are another major class of lipids that have been extensively studied in the context of inflammation and atherosclerosis. Their biology is also complicated and they initiate pro-inflammatory responses in a context-dependent manner. The transcriptome and functional phenotype of BMDMs exposed to the oxidation products of 1-palmitoyl-2-arachidonoyl-sn-3-glycero-phosphorylcholine (oxPAPC) is markedly different to that generated by LPS + IFNγ (M1) and IL-4 (M2) polarization stimuli (Kadl et al., 2010). These MOs, termed Mox, express a narrow range of pro-inflammatory markers (Il-1β, Cox-2, Cxcl2, Cxcl1) (Kadl et al., 2010, 2011), though the amount of Il-1β released is considerably less than from M1 MOs (Kadl et al., 2010), however, this is commensurate with the typically lower levels of inflammation seen in chronic diseases. Tlr2^−/−^ mice were used to confirm the OxPAPC receptor (Kadl et al., 2011). Interestingly, the inflammatory bioactivity was contained in the “long chain” fractions of OxPAPC, but not the short chain fractions or individual oxidized phospholipid constituents POVPC, PGPC, lysoPC (all short chain). Using heme oxygenase-1 (HO-1) as the Mox phenotype-defining marker, this population accounted for a third of all the MOs found in the aortas of LDLR^−/−^ mice following a 30-week “atherogenic” diet (Kadl et al., 2010). The subsequent discovery that an anti-inflammatory subtype, termed Mhem (Boyle et al., 2012), also highly expresses Hmox-1 may indicate a lower abundance of Mox MOs in those mice. Furthermore, there may be undiscovered novel or transient phenotypes that express HO-1.

An intriguing pair of recent studies has further uncovered the complexities of oxPAPC bioactivity. Both reports are shaped around the release of OxPAPC from dying cells and how this affects adjacent cells, and whilst neither explicitly discusses atherosclerosis, their findings are applicable to the plaque microenvironment. Firstly, mouse DCs briefly primed with LPS then stimulated with OxPAPC, release Il-1β in an Nlrp3-dependent manner (Zanoni et al., 2016). When stimulated with either ligand in isolation, this cytokine is not released. This combined LPS/OxPAPC treatment induces something the authors describe as a “hyperactive state” where Il-1β release occurs in the absence of cell death. Active components of OxPAPC (KOdiA-PC, POVPC, PGPC) also induced Il-1β release in DCs, but not in isolation (LPS priming was required). Different forms of OxPAPC were also used, a commercially purchased form and a form the authors synthesized to be enriched in PEIPC, a known bioactive constituent of OxPAPC, and both induced Il-1β release to the same extent. Intriguingly, OxPAPC could not induce Il-1β release in MOs, whether primed or not (Zanoni et al., 2016). The same group further explored this final observation in a subsequent report. Here, they showed that the minor components (< 10%) of OxPAPC, PGPC, and POVPC, are able to hyperactivate LPS-primed MOs, inducing Il-1β release, but OxPAPC itself cannot (Zanoni et al., 2017). As with DCs, the priming signal is essential for cytokine release. This study also identified CD14 as an OxPAPC receptor, whose endocytosis leads to OxPAPC delivery, hyperactivation, and Il-1β release.

A fascinating aspect of OxPL bioactivity is its ability to simultaneously induce inflammation and inhibit LPS signaling through TLR4 and TLR2. This has been known for many years (Bochkov et al., 2002; Walton et al., 2003) and occurs in Mos (Von Schlieffen et al., 2009). Multiple components of the extracellular signaling pathway are inhibited by oxPPLs, including CD14, LPS binding protein and MD-2 (Erridge, 2009). So what is the relevance of this *in vivo*? A subsequent study addressed this by demonstrating that oxPLs are far more potent at inhibiting LPS-mediated inflammation than their own ability to induce it (Oskolkova et al., 2010). This study examined a range of oxPLs aswell as components of oxPAPC (POVPC, PGPC, PEIPC) with all eliciting similar responses in human endothelial cells (HUVECs). Mice injected with LPS and/or oxPAPC replicated the trend observed *in vitro* (Oskolkova et al., 2010). Components of oxPAPC were present at significantly higher concentrations in the atheroma than in the plasma of humans and mice, so given their more prominent pro-inflammatory function at high concentrations, they may exacerbate the inflammatory plaque microenvironment. This of course relies upon their ability to induce release in the absence of a priming signal, which, except for the oxPAPC-induced Il-1β release seen in MOs (Kadl et al., 2010), was not apparent in the other studies referenced here. There may be other TLR4 ligands in the plaque microenvironment that fulfill this function or subclinical endotoxemia, known to aggravate atherosclerosis may provide this stimulus.

### Cholesteryl esters

The diversity and sheer number of lipid species present *in vivo* alongside the difficulty in identifying some of them before refined methods became available, has inevitably meant some lipids have only recently been found whilst others have received little attention. A good example of this are oxidized cholesteryl ester (oxCE) molecules, which have only come under greater scrutiny over the past decade. They are abundantly present in oxLDL and are bioactive, inflammatory components of mmLDL, able to induce Cxcl2 release in mouse MOs (Harkewicz et al., 2008). Using High Performance Liquid Chromatography (HPLC) coupled with tandem Mass Spectrometry, a large and diverse collection of oxidized CEs, many of which were novel, were detected in human atheromata samples relatively recently (Hutchins et al., 2011; Choi et al., 2013). This study uncovered a greater abundance and diversity of oxCE species than had been previously believed to exist. From a therapeutic point of view, understanding the inflammatory capacity of oxCEs is a coveted aim and whilst some studies exist, they are currently thin on the ground. Amongst the extensively oxCEs to have been identified, is cholesteryl (9,11)-epidioxy-15-hydroperoxy-(5Z,13E)-prostadienoate (BEP-CE), produced following oxidation of cholesteryl arachidonate with 15-lipoxygenase. This highly oxygenated cholesteryl ester was shown to be pro-inflammatory in mouse BMDM. It induced Tlr4 dimerization and signaling and the release of Cxcl2. In Syk^−/−^ and Tlr4^−/−^ mice, cytokine release was abolished (Choi et al., 2013). Cholesteryl esters hydroperoxides are abundantly present in oxLDL and are bioactive, inflammatory components of mmLDL, able to induce Cxcl2 release in mouse MOs (Harkewicz et al., 2008). By contrast; 9-oxononanoyl cholesterol (9-ONC), an oxoester from cholesteryl linoleate identified in human atherosclerotic lesions (Hoppe et al., 1997), is not. It is derived from cholesteryl linoleate, upregulates the anti-inflammatory cytokine TGFβ in J774A.1 MOs (Sottero et al., 2005), and increases the expression and protein synthesis of TGFβ and the TGFβ receptor in human U937 promonocytic cells (Gargiulo et al., 2009). Despite these findings, both 9-ONC studies only examined TGFβ and presented no evidence of pro-inflammatory marker quantification. Another oxCE identified in oxLDL (Kobayashi et al., 2001) but which has received little attention is 7-Ketocholesteryl-9-carboxynonanoate. The structure of this lipid only differs from 9-ONC (Boechzelt et al., 1998) by two additional oxygen atoms, yet it induces a range of transcripts in J774A.1 MOs that are consistent with a pro-inflammatory role. These include IL-1R1, ICAM-1, CCR2, and NF-κB, although the PCR array used in this experiment also showed upregulated IL-4 and did not show a significant increase in the expression of several inflammatory cytokines and chemokines, including Il-1β (Huang et al., 2010).

Research in our laboratory has focused on the biology of a novel class of stable end products of CE oxidation named cholesteryl hemiesters (Estronca et al., 2012; Domingues et al., 2017). The only commercially available member of this family, cholesteryl hemisuccinate, induces pro-inflammatory cytokine (Il-1β, Il-6, Tnfα) release in BMDM, but does not affect Il-10 levels relative to native LDL controls (Domingues et al., 2017).

Like many of the lipid classes discussed above, exposure to a low dose of LPS was required for cytokine release. In zebrafish on a Chems-enriched diet, the number of myeloid cells that infiltrate the vasculature is significantly higher than in control or cholesterol-fed fish (Domingues et al., 2017). It can be argued, correctly, that Chems is not a major natural product of LDL oxidation. However, it may be expected that the major products would have the same effects as Chems (ongoing work).

## Fundamental differences in the experimental approaches used to study lipid-mediated inflammation can lead to alternative outcomes

The broad spectrum of lipid classes and products used to model atherogenic, proinflammatory immune responses has facilitated a greater understanding of some of the underlying molecular mechanisms. The field is now awash with data, yet there are emerging concepts that warrant an even deeper understanding in the hope that improved, and perhaps personalized, therapies can be developed to reduce the inflammatory burden, thereby limiting the pathogenesis of atherosclerosis. It is clear from the literature that multiple alternative approaches are often being used to answer the same or similar questions. A good example of this is the different reagents that are used to generate MOs, with either MO Colony-Stimulating Factor (M-CSF) or L-Cell Conditioned Media (LCCM; which contains M-CSF) or Granulocyte-MO Colony-Stimulating Factor (GM-CSF) added to human Mo or mouse bone marrow cultures. This is important because all three of these cytokine options produce MOs with markedly different transcriptomes, including differentially expressed inflammatory and lipid handling genes. Studies carried out a decade ago or earlier showed that human Mos differentiated with GM-CSF generate pro-inflammatory (M1-like) MOs, whilst those exposed to M-CSF develop an anti-inflammatory (M2-like) phenotype (Verreck et al., 2004; Xu et al., 2007; Waldo et al., 2008). A similar trend was also observed in mouse BMDM (Fleetwood et al., 2007, 2009). A comprehensive assessment of the transcriptomes of GM-CSF and M-CSF differentiated human blood Mos and mouse bone marrow was more recently undertaken using microarrays and qPCR (Lacey et al., 2012). Comparing the expression levels of TNFα, IL-1β, IL-12p35, IL-12p40, IL-23p19, IL-8, CCL2, and IL-10 between GM-CSF and M-CSF-differentiated human MOs revealed the only transcripts significantly different between the two populations were CCL2 and IL-10, both elevated in M-CSF-derived MOs (Lacey et al., 2012). Significant differences were observed between the transcriptomes of GM-CSF and M-CSF-derived mouse MOs. Amongst the top 100 most differentially expressed genes, the relative expression levels of several cytokine and chemokine receptors, some chemokines and notably Il-1b and Vcam1 were substantial. The latter two are particularly relevant in atherosclerosis. Strikingly, only 17% of the genes differentially regulated by human GM-CSF and M-CSF had a conserved pattern of transcriptional control in the mouse MOs, suggesting that observations in mouse BMDM may not necessarily translate to human cells. Interestingly, the same group showed in an earlier study that type I IFNs, known to be present in LCCM (Warren and Vogel, 1985), grossly affect the basal transcript levels of a number of genes, including some pro-inflammatory chemokines, expressed in MOs differentiated with M-CSF (Fleetwood et al., 2009), i.e., the CSF in LCCM.

As a master regulator of inflammation, IL-1β bioactivity fundamentally affects atherosclerosis as discussed already. A very recent study has revealed some stark, CSF-dependent differences in how and when IL-1β is produced by human MOs, which have implications for atherosclerosis research *in vitro* (Budai et al., 2017). In it, both GM-CSF and M-CSF-derived MOs release similar amounts of IL-1β, yet the release kinetics are dissimilar. M-CSF-differentiated MOs stimulated with LPS and either ATP or nigericin release a high concentration of IL-1β after just 2 h, which declines substantially after 6 h, returning to baseline levels after 12 h (Budai et al., 2017). By contrast, when GM-CSF was used, IL-1β release was more gradual, peaking between 6 and 24 h yet continuing to remain well above baseline at this latter time point. Perhaps most intriguing of all was the observation that only a priming signal, in this case LPS, was required for release in GM-CSF-polarized MOs. The authors demonstrated IL-1β release in the absence of “signal 2,” and constitutive caspase-1 activity in these cells (Budai et al., 2017). This phenomenon has previously only been shown in Mos (Netea et al., 2009).

Another potentially critical factor in understanding the inflammatory response to lipids is the *type* of MOs that are used. It is apparent in the literature, and clear from the references cited in this article, that both pMOs and BMDMs are used to characterize lipids *in vitro*. Alternative MO phenotypes have distinct functional characteristics yet the differences that may exist between these populations are not usually acknowledged or examined. Based on this logic, a recent study found that when comparing pMO relative to BMDM, in either untreated MOs (basal expression) or oxLDL-transformed foam cells, substantial differences in cell surface marker, and inflammatory cytokine gene expression levels were evident (Bisgaard et al., 2016). Collectively these data expose major differences between the alternative MO populations that are used to study lipid-mediated inflammation. Most *in vitro* studies into lipid and lipoprotein-mediated inflammation use one of five alternative *ex-vivo* MO types. Differentiation of blood or bone marrow-derived Mos produces three of these types in response to either L-cell conditioned media (containing M-CSF + TypeI IFNs) or M-CSF or GM-CSF. Each of these types possess a distinct transcriptional phenotype that has some overlap with the other two CSF-derived types (Fleetwood et al., 2007, 2009; Lacey et al., 2012). Small and large peritoneal MOs are also used for *in vitro* studies. They are transcriptionally distinct from one another and from their bone marrow-derived counterparts. When any of these five types of MOs are exposed to the same exogenous signal, it leads to their polarization, producing novel phenotypes that differ from one another as per the original differentiation signals.

In addition to primary cells, there are many MO cell lines used to study lipid-mediated inflammation, and some of them are perhaps unsuitable. Whilst cell lines offer a more homogenous population, most of them are already polarized toward a particular phenotype. This is important as these cells are almost certain to respond differently to a given lipid compared with a more naive population such as M0 primary MOs. Furthermore, beginning an experiment with polarized cells, e.g., with an M1-like phenotype, isn't very physiological as Mos recruited to the intima first differentiate into M0 MOs followed by polarization according to the signals received from the microenvironment. Some cell lines have also been shown to lack specific genes, have low expression levels of key molecules or have certain signaling pathways switched off. A good example here is the RAW MO cell line that lacks the intracellular inflammasome component ASC (Pelegrin et al., 2008) and is therefore unable to release Il-1β. This is significant because of the profound link between lysosome/mitochondrial dysfunction, metabolism, and inflammation (Guo et al., 2015; Van Den Bossche et al., 2017). These might seem like easy and obvious statements to make; however there continue to be reports describing a particular lipid inducing a particular effect, yet, the experimental conditions used can sometimes seem questionable.

Other experimental parameters are subject to variation and can be critical when studying inflammation. One example is the vehicle used to deliver a lipid. This is illustrated rather well with 7-ketocholesterol (7KC), which is found abundantly in oxLDL. Feeding the U937 Mo line either with 7KC or oxLDL induces a loss of cell viability. Both also cause glutathione loss and lead to increased 7KC content in these cells. By contrast, when acLDL is used to deliver this sterol to U937s, its uptake is high yet the toxicity to U937 cells is very low (Rutherford and Gieseg, 2012). Another example is the use of serum in cellular assays. In response to oxPAPC stimulation, one study showed that serum components were essential for the expression of inflammatory mediators IL-1β and COX-2, but not for HO-1 in BMDM (Kadl et al., 2011). Serum starvation is a commonly employed procedure *in vitro*, however, it has the potential to alter the outcome of an experiment (Pirkmajer and Chibalin, 2011). It may therefore be prudent when studying lipid-driven inflammation to always work with both serum-free and cultures with serum present if there is a need or desire to examine serum-starved cells.

The precise composition of lipid mixtures used to characterize inflammation also seems to vary greatly. For instance, with a complex mixture such as LDL, variable standards and methodologies of oxidation are employed. These include the use of 12/15-lipoxygenase (LO), copper, or osmium tetraoxide amongst others and have been reviewed extensively elsewhere (Levitan et al., 2010; Tsimikas and Miller, 2011). With no single or standard method of derivatization, some variation in the bioactivity of certain oxidized lipids have been reported. No two batches of oxLDL can be exactly alike, and it is often the case that post-oxidation analysis of a lipid mixture is not carried out. So in some studies, the exact composition of what is being added to the cells is not known. Given the array of immunogenic species that exist within oxLDL, with variable amounts of oxidized components likely between batches, this is certain to affect the intensity of pro- or anti-inflammatory responses in MOs or other cells. Consequently, there is a spectrum of reported inflammatory phenotypes for commonly studied lipid mixtures. This makes deciphering the impact upon other critical related parameters, such as immunometabolism, difficult. It is important to note there are many excellent examples of groups and studies (Harkewicz et al., 2008; Kadl et al., 2010; Oskolkova et al., 2010; Van Der Vorst et al., 2017; Zanoni et al., 2017) that employ stringent, robust methodologies. It would be greatly beneficial if some form of standardization or guidelines, in line with studies such as these, could be introduced.

Several of the studies reviewed in this article have highlighted the benefit of assessing the function of individual lipid components. The most striking of these perhaps being where oxPAPC and its minor fractions hyperactivate DCs to release IL-1β, however, only the minor fractions activate MOs (Zanoni et al., 2017). All of these reports make a strong case for refining the way *in vitro* experiments are carried out, by moving away from using complex mixtures and instead switching to individual lipid species or low complexity mixtures enriched with a single lipid component. In doing so, it is going to become easier to associate specific effects with specific lipid structures, which will improve the development of both generic and personalized therapies aimed at reducing or controlling atherosclerosis and other inflammatory diseases.

## Therapeutic interventions to treat atherosclerosis

By restoring or augmenting cellular function, or by inhibiting the factors that disturb it, numerous therapeutic interventions have been developed to treat atherosclerosis. Anti-inflammatory therapies are particularly ubiquitous and have a range of targets, with variable levels of efficacy. These include nutraceuticals, which are ingested as dietary supplements and have shown beneficial reductions in ROS activity, endothelial exocytosis, MO migration, and promote reverse cholesterol transport (Moss and Ramji, 2016). 3-Hydroxy-3-methylglutaryl coenzyme A (HMG-CoA) reductase inhibitors, also termed statins are the most extensively used class of cholesterol lowering drugs that specifically target the mevalonate pathway. They were also recently shown to significantly and continuously suppress plaque inflammation by inhibiting MO proliferation (Tang et al., 2015). The use of cyclodextrin has also shown considerable benefit, through regression of plaque size. It increases the solubility of cholesterol, reducing the damaging effects of cholesterol crystals; and through targeting a gene set under the control of liver-X-receptors, promotes cholesterol efflux and anti-inflammatory effects (Zimmer et al., 2016). Epigenetic reprogramming can augment inflammation in trained immune cells and whilst atherosclerosis-specific therapies are not currently available to remodel methylated DNA, histone deacetylase inhibitors offer a potential novel option to reduce inflammation and are being experimented in some preliminary studies (Christ et al., 2016). A long standing and gradually improving treatment option is to directly block cytokines or the NLRP3 inflammasome, using either drugs or monoclonal antibodies (Toldo and Abbate, 2018). Controlling the biology of Interleukin-1 beta (IL-1β), which drives the progression of atherosclerosis (Libby et al., 2014; Libby and Hansson, 2015), leading to cardiovascular complications, has been of particular interest. A large-scale clinical trial (named CANTOS) that ended in early 2017 aimed to directly gauge the influence of IL-1β on atherothrombosis (Ridker et al., 2017). Enrolled patients received quarterly doses of canakinumab, a monoclonal antibody directed against IL-1β, for 3 years. Clinical outcomes included a significant decrease in non-fatal myocardial infarction, in non-fatal stroke and in cardiovascular death. These general results were underpinned by considerable reductions in selected inflammatory biomarkers C-reactive protein and IL-6, however, HDL and LDL levels were unaffected whilst triglycerides rose slightly (Ridker et al., 2017). The success of this trial reinforced the theory that IL-1β-mediated inflammation is a major driver of this disease. Blocking just a single molecule is unlikely to restrict all of the signaling pathways that lead to the production of additional inflammatory mediators. Other members of the IL-1 family including IL-1α (Freigang et al., 2013), IL-18 (Wang et al., 2015) as well as IL-6 (Schieffer et al., 2004) and TNFα (Brånén et al., 2004) are known to participate in atherogenesis and offer attractive future targets. PCSK9, a serine protease that binds to the extracellular domain of the LDL receptor targeting it for lysosomal degradation, also mediates inflammation in atherosclerotic plaques, including the synthesis of pro-inflammatory cytokines such as IL-1α, IL-6, and TNFα (Wicinski et al., 2017). A monoclonal antibody to PCSK9 (evolocumab) was recently used in a clinical trial (Sabatine et al., 2017), which led to substantial reductions in LDL cholesterol levels and the incidence of cardiovascular events. No data was presented on the impact upon inflammation, but it is highly likely to have been reduced, contributing to the improved clinical outcomes. Finally, a small but growing number of studies are exploring the benefits of restoring lysosome function through biogenesis. The activation of lysosome biogenesis by TFEB overexpression suppresses cholesterol crystal-induced NLRP3 inflammasome, attenuating the progression of atherosclerosis, *in vitro* and *in vivo* (Emanuel et al., 2014). More recently, TFEB overexpression was shown to reverse the gradual decline in the autophagy–lysosome system that occurs in lipid loaded cells with dysfunctional lysosomes, abolishing apoptosis and reducing IL-1β production, and consequently atherosclerosis (Sergin et al., 2017).

## Summary

The purpose of this review was to offer a perspective on how cell dysfunction and inflammation contribute to atherosclerosis. We described how critical changes in organelle function, particularly those affecting lysosomes, can transform the inflammatory status of macrophages, the most prominent cell type in atherogenesis and which has a major influence on lesional inflammation, plaque progression and stability. By discussing lipid and non-lipid factors that can regulate inflammation, including the perhaps under appreciated role of circadian rhythms, we outlined some probable targets for more precise therapeutic interventions. Of course a major area of modern biomedical research is understanding the biology of lipid and lipoprotein factors that contribute to atherosclerosis and other chronic diseases, and some of the more prominent compounds studied were reviewed. In carrying out such studies, there are evident discrepancies in the literature, that in many cases are caused by alternative approaches to asking the same question. We made a point of highlighting where we believe these problems exist and hope that some form of standardization or guidelines could be introduced into the field to address them. Finally, we discuss recent advances in generic treatment options for atherosclerosis, which ideally could form one aspect of more personalized future novel therapies.

## Author contributions

All of the authors listed were involved in the preparation of the manuscript. ND carried out some of the experimental work referred to in the passages describing data produced in our group.

### Conflict of interest statement

The authors declare that the research was conducted in the absence of any commercial or financial relationships that could be construed as a potential conflict of interest.
